# Synovial histopathology in rheumatoid arthritis treated with biological disease-modifying antirheumatic drugs: an analysis of 1593 surgical specimens using the Rooney score

**DOI:** 10.1016/j.ero.2026.02.008

**Published:** 2026-03-10

**Authors:** Masanori Sudo, Asami Abe, Hajime Ishikawa, Sayuri Takamura, Daisuke Kobayashi, Kiyoshi Nakazono, Akira Murasawa, Suguru Yamamoto, Satoshi Ito

**Affiliations:** 1 Department of Rheumatology, Niigata Rheumatic Center, Niigata, Japan; 2 Division of Clinical Nephrology and Rheumatology, Kidney Research Center, Niigata University Graduate School of Medical and Dental Sciences, Niigata, Japan

## Abstract

**Objectives:**

This study aims to evaluate synovial histopathological inflammation in rheumatoid arthritis (RA) using the Rooney score (RS) and to investigate histopathological heterogeneity within difficult-to-treat (D2T) RA.

**Methods:**

We analysed 1593 synovial specimens from 1043 patients with RA who underwent joint surgery between 2013 and 2023. Histological inflammation was assessed by a blinded pathologist using a modified RS (total score, 0-60). Patients were classified as biological disease-modifying antirheumatic drug (bDMARD)-naïve or bDMARD-treated; the treated group was further subdivided into D2T and non-D2T RA according to the 2021 European League Against Rheumatism definition. Associations between RS and clinical markers, including C-reactive protein (CRP), Disease Activity Score 28–erythrocyte sedimentation rate (DAS28-ESR), matrix metalloproteinase-3 (MMP-3), and power Doppler (PD) grade, were examined. Exploratory analyses further stratified D2T RA into inflammatory and noninflammatory subtypes based on objective inflammatory markers.

**Results:**

RS was significantly lower in bDMARD-treated patients than in bDMARD-naïve patients, indicating histological attenuation of synovial inflammation. Item-wise analysis showed lower scores for synoviocyte hyperplasia and lymphocytic infiltration and higher fibrosis scores in the treated group. Total RS correlated with CRP, DAS28-ESR, MMP-3, and PD grade. Among bDMARD-treated patients, RS did not differ between D2T and non-D2T RA, whereas MMP-3 levels were higher in D2T RA. In exploratory analyses, inflammatory D2T RA showed higher RS and MMP-3 levels than noninflammatory D2T RA.

**Conclusions:**

bDMARD therapy attenuates synovial inflammation histologically, and RS reflects local inflammatory activity. Exploratory analyses suggested that the synovial histopathology may provide complementary information on inflammatory heterogeneity within D2T RA, which may not be fully captured by the conventional clinical indices.

**WHAT IS ALREADY KNOWN ON THIS TOPIC**

• Synovial histopathology reflects inflammatory activity in rheumatoid arthritis.
• Biological disease-modifying antirheumatic drugs (bDMARDs) improve clinical disease activity in RA.

**WHAT THIS STUDY ADDS**

• In a large cohort of 1,593 synovial specimens, Rooney scores were significantly lower in bDMARD-treated patients than in bDMARD-naïve patients.
• Synovial histopathology revealed heterogeneity within difficult-to-treat RA.

**HOW THIS STUDY MIGHT AFFECT RESEARCH, PRACTICE OR POLICY**

• Synovial histopathology may provide complementary information to clinical indices when evaluating inflammatory activity and heterogeneity in RA.

Alt-text: Unlabelled box dummy alt text

## INTRODUCTION

Rheumatoid arthritis (RA) is a chronic systemic autoimmune disease characterised by persistent synovitis, progressive joint destruction, and systemic complications. The introduction of biological disease-modifying antirheumatic drugs (bDMARDs) has dramatically improved the clinical management of RA, enabling many patients to achieve remission or maintain low disease activity through early and targeted therapeutic strategies [[1], [2], [3]]. Consequently, radiographic progression has slowed, physical function has improved, and quality of life has increased in a significant proportion of patients [4].

Despite these remarkable clinical advances, histopathological evaluation of synovial tissue, the site of inflammation, lags behind routine assessments. The extent to which bDMARD therapy ameliorates synovial inflammation at the local tissue level has not yet been fully elucidated. Most clinical studies have focused on composite indices such as the Disease Activity Score 28 (DAS28) or imaging modalities such as ultrasound and magnetic resonance imaging, whereas direct pathological evidence of the therapeutic response remains scarce [4,5].

Histopathological analysis provides unique insights into the immunopathology of RA by capturing features, such as synoviocyte hyperplasia, vascular proliferation, and lymphoid infiltration. Among the available grading systems, the Rooney score (RS), proposed in 1988, is one of the most widely adopted semiquantitative methods for assessing RA synovitis [6]. Previous work by Abe et al [7] demonstrated that RS correlates with power Doppler (PD) ultrasound signals and that tumour necrosis factor (TNF) inhibitor treatment reduces specific RS components such as synovial lining hyperplasia and lymphocytic infiltration. These findings suggest that RS can serve as a meaningful histopathological marker of the therapeutic response.

Nevertheless, large-scale studies evaluating the histological effects of bDMARDs in real-world cohorts are limited. Moreover, the pathological features of difficult-to-treat (D2T) RA, a subgroup defined by the European League Against Rheumatism (EULAR) criteria as having persistent symptoms despite multiple disease-modifying antirheumatic drugs (DMARDs) [8], have not been fully clarified. It is still unclear whether these patients show more severe synovial inflammation histologically or whether bDMARD therapy remains effective at suppressing local tissue-level inflammation, even in clinically refractory cases.

D2T RA is increasingly recognised as a heterogeneous condition in which persistent inflammatory activity may coexist with noninflammatory mechanisms such as structural damage and pain sensitisation. Tissue-based approaches, including synovial pathotyping and biopsy-driven studies, have highlighted the potential role of synovial assessment in therapeutic stratification beyond conventional clinical indices.

Therefore, the aim of this study was to assess the histopathological effects of bDMARD therapy in the RA synovium using RS in a large set of surgical tissue specimens and to determine whether patients with D2T RA exhibit more severe synovial inflammation than those with non-D2T RA.

## METHODS

This retrospective observational study was conducted at a single tertiary referral centre in Japan and included 1593 synovial tissue specimens obtained from 1043 patients diagnosed with RA who underwent joint surgery between 2013 and 2023. All patients met the 2010 American College of Rheumatology/EULAR classification criteria for RA [9].

Synovial specimens were collected, formalin-fixed, dehydrated, embedded in paraffin, processed, and stained with haematoxylin and eosin according to standard histopathological protocols. Histological inflammation was assessed using a modified RS, based on the original scoring method proposed by Rooney et al [6]. This system evaluates 6 histological features, synoviocyte hyperplasia, fibrosis, proliferating blood vessels, perivascular infiltrates of lymphocytes, focal aggregates of lymphocytes, and diffuse infiltrates of lymphocytes, each scored on a 0 to 10 scale across 3 representative high-power fields, yielding a total score ranging from 0 to 60. Histological scoring was performed by a single experienced pathologist who was blinded to clinical information.

Patients were classified into 2 main groups based on their treatment history: those who had never received bDMARDs (bDMARD-naïve group) and those who had been treated with 1 or more bDMARDs (bDMARD-treated group). The latter was further divided into 2 subgroups according to the 2021 EULAR definition of D2T RA [8], which requires the failure of at least 2 biological or targeted synthetic DMARDs and the presence of ongoing disease activity. To address the potential heterogeneity within D2T RA, exploratory analyses were performed by stratifying D2T RA into inflammatory and noninflammatory subtypes. Inflammatory D2T RA was defined by the presence of objective evidence of active inflammation at the time of surgery, based on at least one of the following criteria: C-reactive protein (CRP) >0.3 mg/dL, DAS28-erythrocyte sedimentation rate (ESR) ≥3.2, or PD grade ≥1 in the operated joint. Patients who did not meet any of these criteria were classified as having noninflammatory D2T RA. This operational definition was intended to reflect the real-world clinical decision-making process at the time of surgery, and it was not designed to establish a definitive biological classification. These analyses were conducted in an exploratory manner and were intended to complement, rather than replace, the primary comparisons between D2T and non-D2T RA. Thus, comparisons were made among the 3 groups: bDMARD-naïve, non-D2T RA, and D2T RA.

Clinical data at the time of surgery were extracted from medical records, including patient demographics (age, sex, and disease duration), current or past use of glucocorticoids and conventional synthetic DMARDs, levels of CRP, matrix metalloproteinase-3 (MMP-3), anticitrullinated protein antibody titres, DAS28-ESR, PD grade on joint ultrasonography, and Larsen grade [10] for radiographic damage. PD grading was performed by experienced rheumatologists using a semiquantitative 0 to 3 scale based on intra-articular vascularity within 1 week before surgery. Preoperative PD assessment was performed only for the joints scheduled for surgery, and the PD score of each operated joint was recorded for the analysis [11].

Additional analyses beyond total RS were performed to further explore the pathological impact of bDMARD therapy. First, each of the 6 items of the RS was individually assessed to determine the histological findings that were most affected by bDMARDs. Second, correlations between the total RS and clinical markers of disease activity (CRP, MMP-3, DAS28-ESR, and PD grade) were examined using Spearman’s rank correlation to evaluate the relationship between histological and systemic inflammation. Finally, a subgroup analysis was conducted to compare histological outcomes across major bDMARD classes: TNF inhibitors (eg, infliximab, adalimumab, certolizumab pegol, etanercept, and golimumab), interleukin-6 (IL-6) receptor inhibitors (eg, tocilizumab and sarilumab), and T-cell costimulation modulators (eg, abatacept).

For classification by bDMARD class, the bDMARD administered immediately before surgery was used. In cases where patients received Janus kinase (JAK) inhibitors before surgery, the classification was based on the most recent biological agent administered before switching to a JAK inhibitor.

The primary analyses were conducted at the specimen level. Because some patients underwent multiple surgical procedures, more than 1 synovial specimen from the same patient was included in the analysis. All statistical analyses were performed using Easy R (EZR) (Saitama Medical Center, Jichi Medical University), a graphical user interface for R (The R Foundation for Statistical Computing). EZR is widely used in clinical research and is based on R with additional functions specific to biostatistics [12]. Continuous variables were summarised as medians with IQRs, and categorical variables were presented as counts and percentages. Between-group comparisons were conducted using the Mann-Whitney *U* test or Kruskal-Wallis test, followed by Steel-Dwass post hoc testing where applicable. Chi-square or Fisher’s exact test was used for categorical variables. Spearman’s rank correlation was used for nonparametric correlation analysis. Two-tailed *P* values of <.05 were considered to indicate statistical significance.

Item-wise analyses of RS components were performed in an exploratory manner, and no formal adjustment for multiple comparisons was applied.

This study was approved by the Institutional Review Board of the Niigata Rheumatic Center (approval no. 2025-020). Informed consent was obtained using an opt-out method approved by the ethics committee.

## RESULTS

A total of 1593 synovial specimens from 1043 patients with RA were included in this study. The bDMARD-naïve group consisted of 1010 specimens and the bDMARD-treated group included 583 specimens.

**Figure 1:**
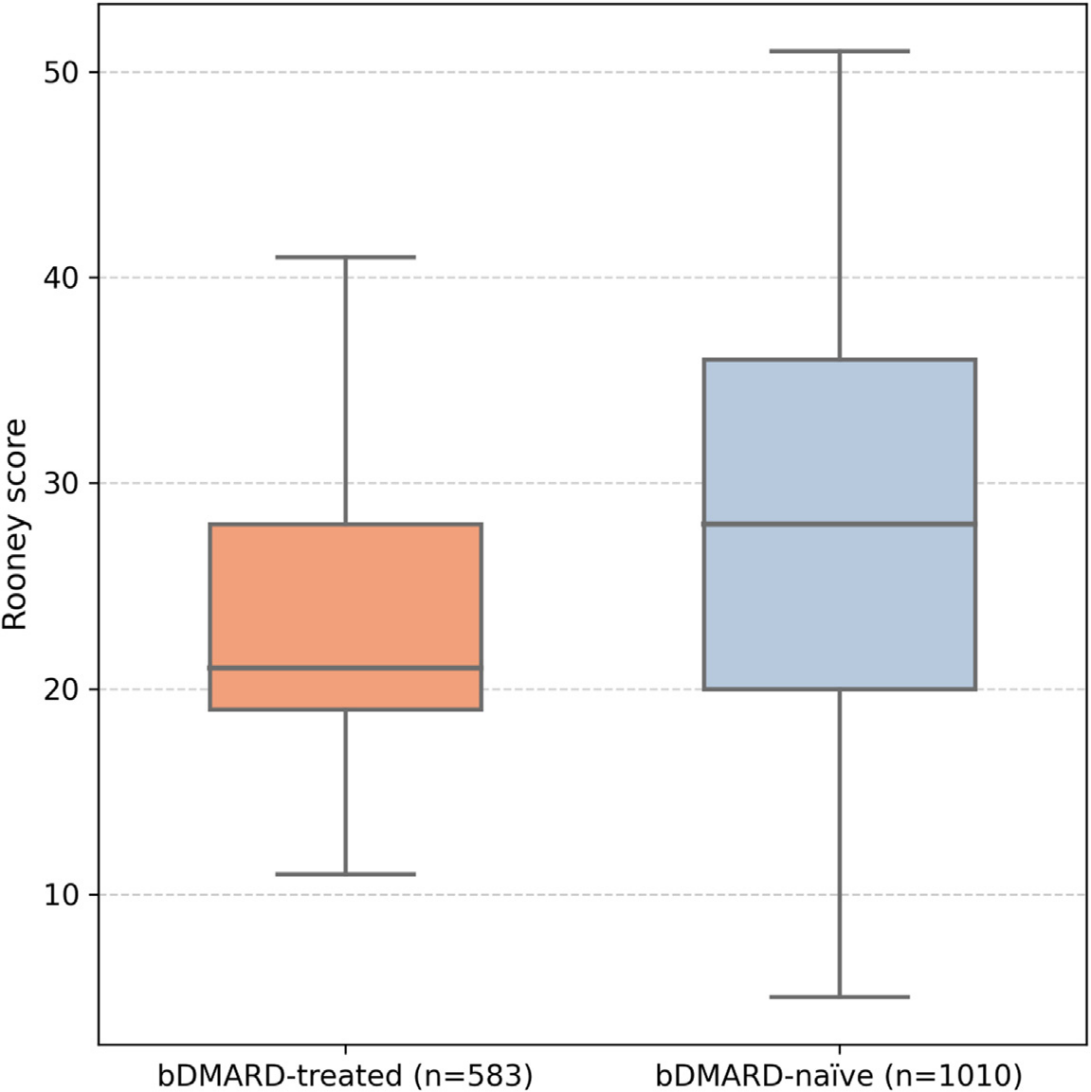
Rooney score by treatment group. Rooney scores were significantly lower in the bDMARD-treated group than in the bDMARD-naïve group (*P* < .0001, Mann-Whitney *U* test). Boxes represent the IQR; horizontal lines indicate the median; and each dot represents an individual synovial specimen. The results of the statistical comparison between the 2 groups are shown by a horizontal bracket and the corresponding *P* value. bDMARD, biological disease-modifying antirheumatic drug.

At the time of surgery, patients in the bDMARD-treated group had significantly lower levels of inflammatory markers and disease activity indices than those in the bDMARD-naïve group. The median CRP was 0.08 mg/dL (n = 583) in the bDMARD-treated group vs 0.26 mg/dL (n = 1010) in the naïve group (*P* < .001). Similar trends were observed for DAS28-ESR and MMP-3 levels. These findings indicate an overall improvement in systemic disease control in the bDMARD-treated group (Table and Fig. 1).

**Table:** Patient characteristics of bDMARD-naïve and bDMARD-treated groups

Item	bDMARD-naïve (n = 1010)	bDMARD-treated (n = 583)	*P* value
Age (y)	68 (60-76)	65 (56-72)	<.001^a^
Sex (female, n [%])	872 (86.3)	523 (89.7)	.059
Disease duration (y)	12 (5-22)	16 (10-23)	<.001^a^
Surgical site (n)	Wrist 356 Finger 219 Elbow 80 Knee 168 Shoulder 11 Ankle 28 Foot 148	Wrist 168 Finger 136 Elbow 43 Knee 94 Shoulder 10 Ankle 26 Foot 106	.057
PSL (mg/d)	1 (0-4)	2 (0-4)	.020^a^
MTX (mg/wk)	4 (0-8)	3 (0-8)	.009^a^
SASP (mg/d)	1000 (1000-1000)	1000 (500-1000)	.759
BUC (mg/d)	200 (100-200)	200 (100-200)	.996
IGU (mg/d)	50 (25-50)	50 (25-50)	.606
TAC (mg/d)	1.5 (1-2)	2.0 (1-3)	.082
MZR (mg/w)	300 (300-450)	450 (300-450)	.245
First bDMARD	NA	IFX 123 ETN 183 ADA 66 GLM 48 CZP 39 TCZ 72 ABT 52	NA
Duration of bDMARD therapy (y)	NA	5 (2-8)	NA
No. of bDMARDs used (n)	NA	1 (1-2)	NA
D2T RA (n, %)	NA	190 (32.6)	NA
PD grade ≥1 (n, %)	830 (82.2%)	416 (71.4%)	<.001^a^
ACPA (U/mL)	100.0 (15.1-300.2)	93.5 (16.2-341.6)	.680
CRP (mg/dL)	0.26 (0.1-0.9)	0.08 (0.0-0.4)	<.001^a^
DAS28-ESR	3.62 (2.80-4.29)	3.16 (2.43-4.04)	<.001^a^
MMP-3 (ng/mL)	107 (69.3-178.6)	87 (54.4-147.0)	<.001^a^
Larsen grade	4 (3-4)	4 (3-4)	.165

ABT, abatacept; ACPA, anti-citrullinated protein antibody; ADA, adalimumab; bDMARD, biological disease-modifying antirheumatic drug; BUC, bucillamine; CRP, C-reactive protein; CZP, certolizumab pegol; DAS28-ESR, Disease Activity Score 28–erythrocyte sedimentation rate; D2T RA, difficult-to-treat rheumatoid arthritis; ETN, etanercept; GLM, golimumab; IFX, infliximab; IGU, iguratimod; MMP-3, matrix metalloproteinase-3; MTX, methotrexate; MZR, mizoribine; PD, power Doppler; PSL, prednisolone; SASP, salazosulfapyridine; TAC, tacrolimus; TCZ, tocilizumab; NA, not applicable.

Values are presented as the median (IQR) or number (%) unless otherwise indicated. *P* values were calculated using the Mann-Whitney *U* test for continuous variables and the chi-square test for categorical variables. Statistical significance was set at *P* < .05.

a Statistically significant (*P* < .05).

**Figure 2:**
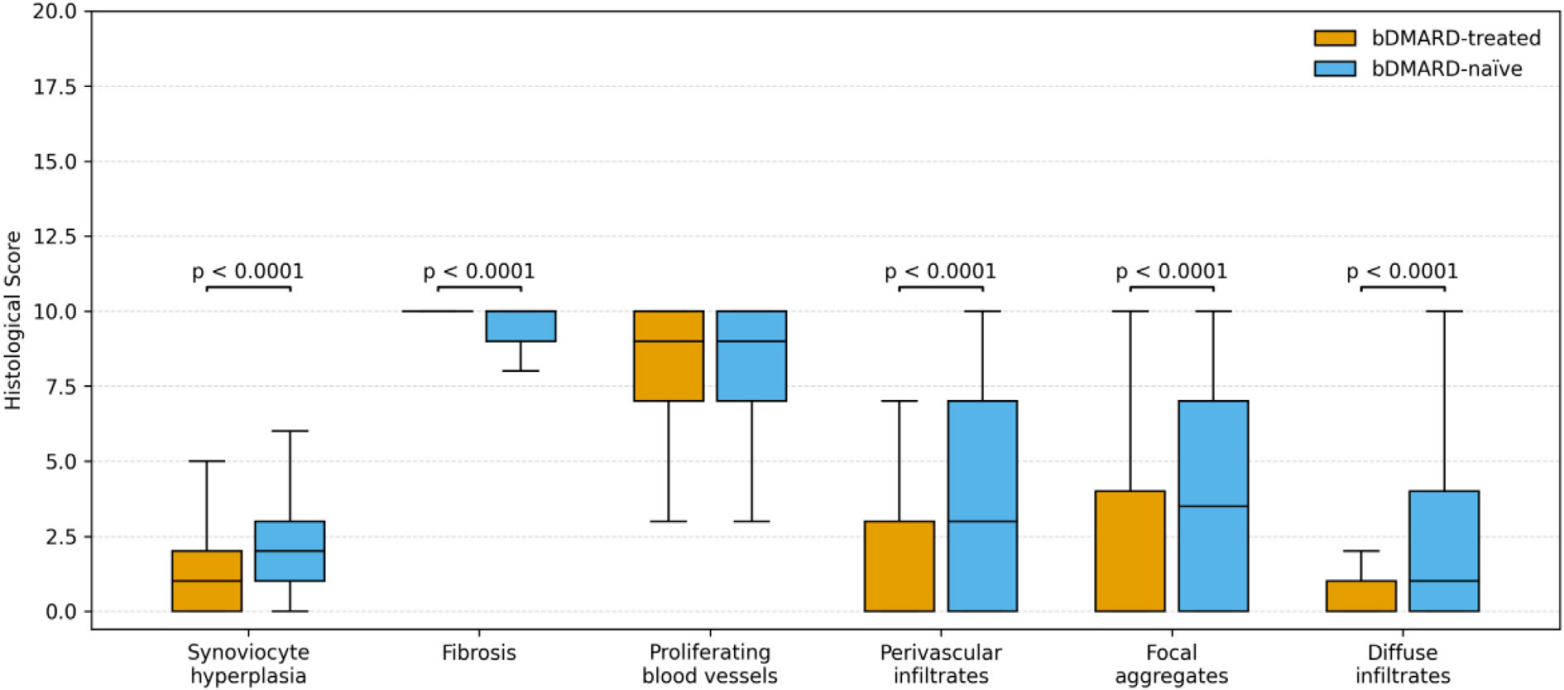
Histological components of the Rooney score by treatment group. Relative to bDMARD-naïve patients, synovial tissues from bDMARD-treated patients showed significantly lower scores for synoviocyte hyperplasia and perivascular and diffuse lymphocytic infiltration, whereas fibrosis scores were significantly higher (*P* < .05). Boxes indicate IQRs, and horizontal lines indicate medians. *P* values represent comparisons between groups using the Mann-Whitney *U* test. Item-wise comparisons were exploratory in nature, and no adjustment for multiple comparisons was applied. bDMARD, biological disease-modifying antirheumatic drug.

The item-wise analysis revealed that the treated group had significantly lower scores for synoviocyte hyperplasia and lymphocytic infiltration, including perivascular, focal, and diffuse aggregates (all *P* < .001). In contrast, fibrosis scores were slightly higher (*P* < .001), whereas no significant differences were observed in proliferating blood vessels (*P* = .34) (Fig 2).

**Figure 3:**
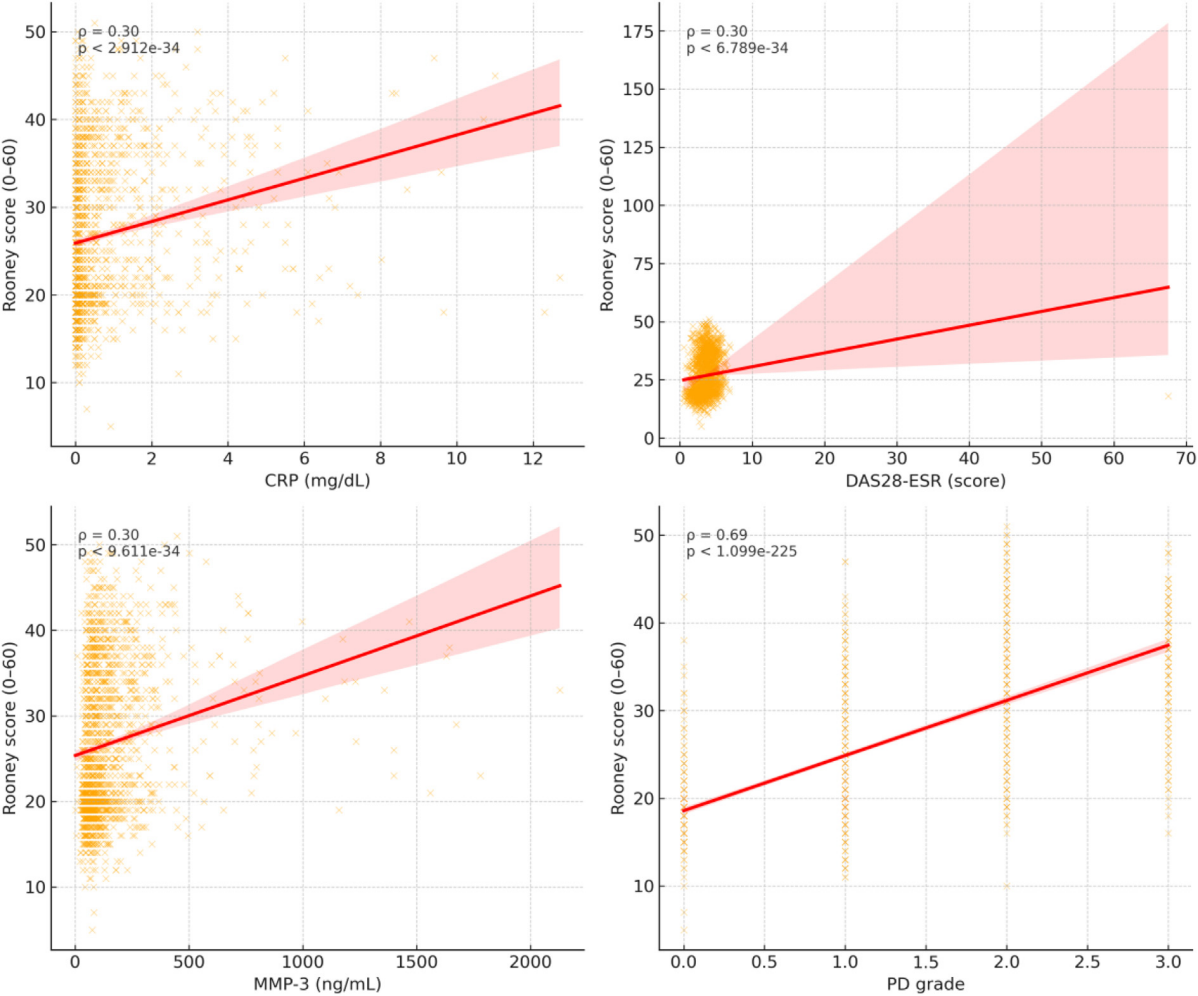
Correlations between Rooney score and inflammatory markers. Scatter plots show the associations between the Rooney score (0-60) and CRP (mg/dL), DAS28-ESR, MMP-3 (ng/mL), and power Doppler (PD) grade (0-3). Spearman’s rank correlation coefficients (ρ) and *P* values are shown in each panel. CRP, C-reactive protein; DAS28-ESR, Disease Activity Score 28–erythrocyte sedimentation rate; MMP-3, matrix metalloproteinase-3.

Correlation analyses showed that the total RS was positively correlated with CRP (ρ = 0.30), DAS28-ESR (ρ = 0.30), MMP-3 (ρ = 0.30), and PD grade (ρ = 0.69) (all *P* < .001) (Fig 3).

The baseline clinical characteristics of bDMARD-treated patients stratified by D2T status are summarised in Supplementary Table S1. There were no significant differences between patients with D2T RA and those with non-D2T RA in terms of age, sex, disease duration, inflammatory markers (CRP, DAS28-ESR, and PD grade), or radiographic damage assessed using the Larsen grade. As expected, patients with D2T RA had a longer duration of biologic therapy and received a greater number of bDMARDs.

**Figure 4:**
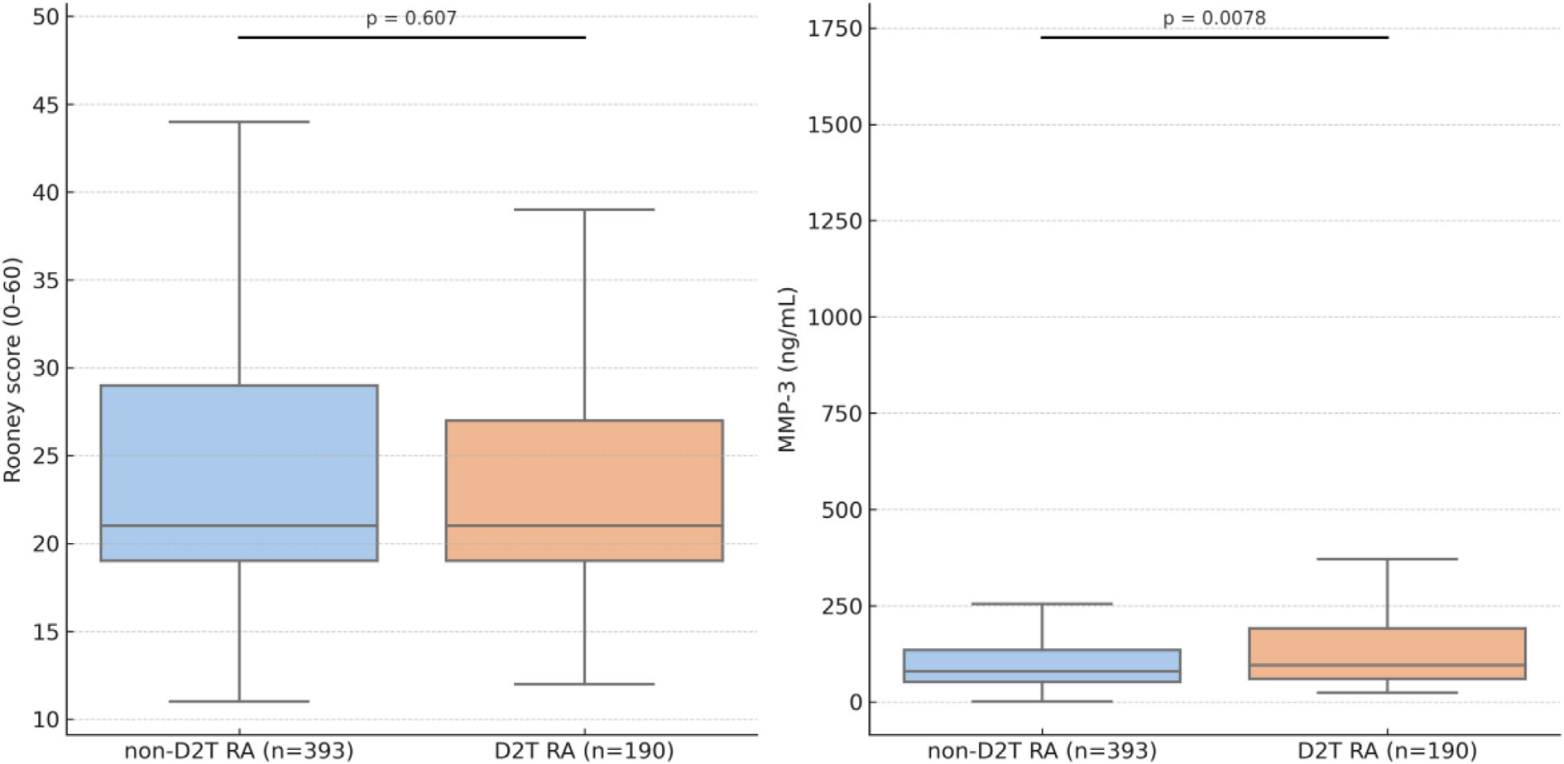
Comparison of histological and biomarker parameters between D2T and non-D2T RA. Box plots show the distributions of Rooney scores and MMP-3 levels in bDMARD-treated patients with D2T RA and non-D2T RA. Boxes indicate IQRs, horizontal lines indicate medians, and dots represent individual specimens. *P* values were calculated using the 2-sided Mann-Whitney *U* test. bDMARD, biological disease-modifying antirheumatic drug; D2T, difficult-to-treat; MMP-3, matrix metalloproteinase-3; RA, rheumatoid arthritis.

In the subgroup analysis of the bDMARD-treated population, RSs showed no significant difference between D2T and non-D2T RA (median 21.0 vs 21.0, *P* = .61). In contrast, MMP-3 levels were significantly higher in the D2T group (median 95.6 vs 79.9 ng/mL, *P* = .008), whereas CRP, DAS28-ESR, PD levels, and prednisolone dose did not differ between the groups (Fig 4).

In exploratory analyses, patients with inflammatory D2T RA showed higher total RSs and MMP-3 levels than those with noninflammatory D2T RA (Supplementary Table S2 and Supplementary Figure S1).

**Figure 5:**
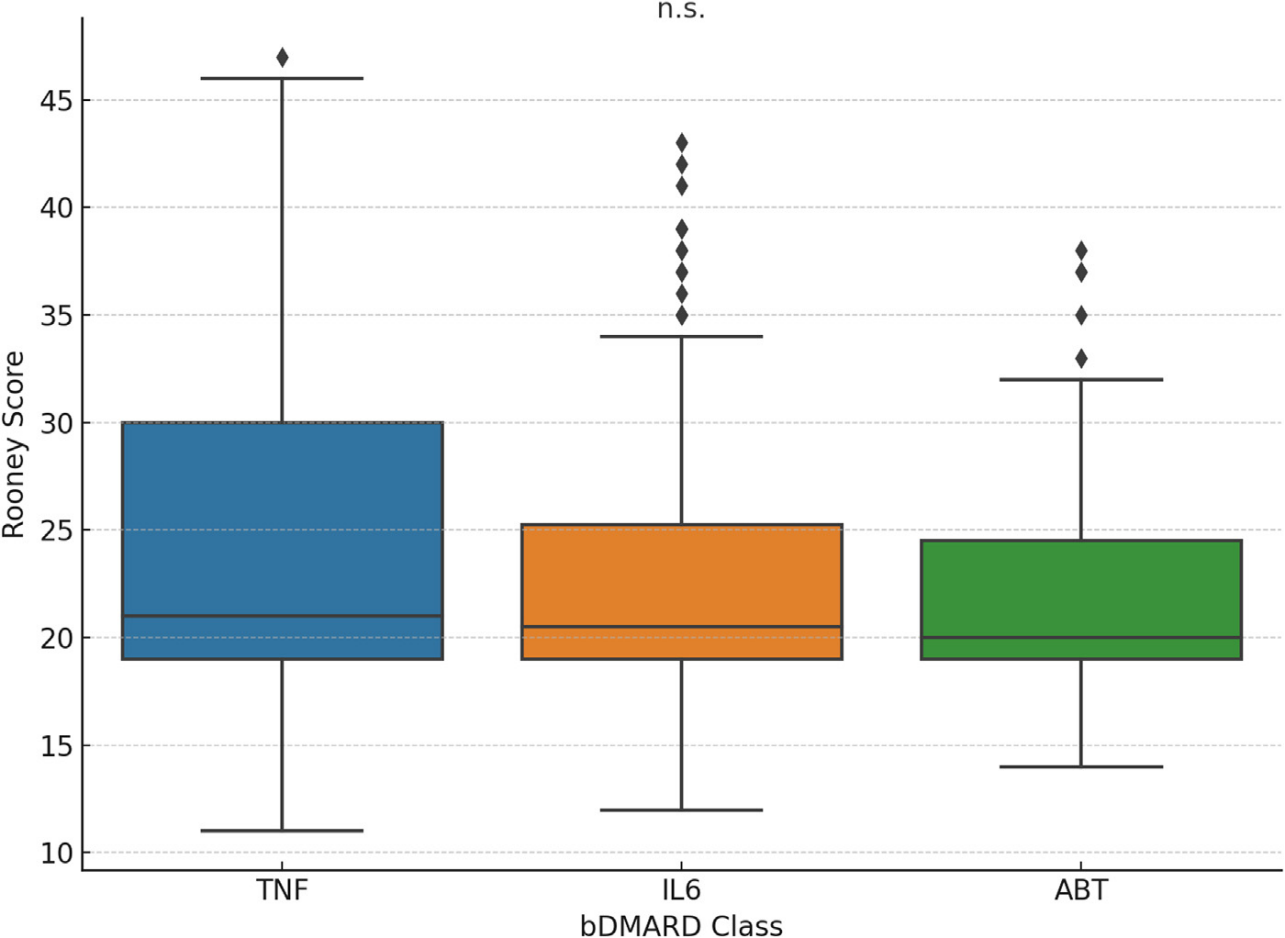
Comparison of Rooney score by bDMARD class. Comparison of Rooney score by bDMARD class (specimen level, n = 583). Patients were categorised, per specimen, according to the most recently administered non-JAK bDMARD before surgery: TNF inhibitors (infliximab, etanercept, adalimumab, golimumab, certolizumab pegol; n = 387), IL-6 receptor inhibitors (tocilizumab, sarilumab; n = 127), and T-cell costimulation inhibitor (abatacept; n = 69). Synovial inflammation tended to be lower in the IL-6 group than in the TNF and abatacept groups, although the differences were not statistically significant (Kruskal-Wallis test, *P* ≥ .05). Rooney scores are presented as box plots with overlaid individual data points. ABT, abatacept; bDMARD, biological disease-modifying antirheumatic drug; JAK, Janus kinase; IL-6, interleukin-6; TNF, tumour necrosis factor.

When comparing histopathological scores across bDMARD classes, patients treated with IL-6 receptor inhibitors tended to have a lower total RS than those receiving abatacept or TNF inhibitors, although the differences were not statistically significant (Fig 5).

## DISCUSSION

Our large-scale histopathological evaluation of synovial tissue in patients with RA undergoing joint surgery demonstrated that bDMARD therapy was associated with a significant attenuation of synovial inflammation at the tissue level. This was reflected by lower total RSs, reduced lymphocytic infiltration, and relative predominance of fibrotic features in the bDMARD-treated group. Notably, active inflammatory features such as synoviocyte hyperplasia, lymphocytic infiltration, and vascular proliferation were markedly diminished, whereas features associated with inactive inflammatory findings, particularly fibrosis, appeared to be relatively increased. These findings suggest a transition from active to chronic inflammation towards tissue repair under the influence of bDMARD therapy [1,2].

Our results were consistent with our previous report showing a reduction in histological synovitis and PD signals following biological therapy [3]. Our study strengthens the utility of histopathological scoring as a complementary measure of treatment response by including a larger and more diverse sample across the bDMARD classes. Moreover, the observed correlations between RS and systemic inflammatory markers such as CRP, DAS28-ESR, MMP-3, and PD grade support the biological relevance of the histopathological assessment of RA [3,6].

Several histopathological scoring systems have been proposed for the assessment of synovitis in RA, including the Krenn score, which is widely used in synovial biopsy-based studies. The Krenn score was originally developed to evaluate active inflammatory synovitis, particularly in patients with early or untreated disease, and it focuses primarily on synovial lining hyperplasia, stromal cellularity, and inflammatory infiltrates. In contrast, the RS provides a broader semiquantitative assessment encompassing not only active inflammatory features, but also chronic changes, such as fibrosis, which are frequently observed in long-standing disease.

Given that the present study exclusively analysed surgically obtained synovial specimens from patients with established RA, often after a prolonged disease duration and multiple therapeutic exposures, we considered the RS to be more suitable for capturing the full spectrum of the histopathological changes in this setting. In particular, the inclusion of fibrotic and structural components allowed us to evaluate the transition from active inflammation to chronic tissue remodelling under biological therapy. Therefore, the use of the RS in this large surgical cohort was a deliberate methodological choice, aligned with the clinical and pathological characteristics of the study population.

In addition, retrospective rescoring of all specimens using the Krenn system was not feasible, as the original pathological evaluations had been performed consistently using the RS as part of routine clinical practice over an extended period. As a result, maintaining methodological consistency across this exceptionally large surgical cohort was therefore prioritised to ensure the internal validity and interpretability of the results.

Contrary to initial expectations, the synovial histopathology at the operated joint did not differ between patients with D2T and non-D2T RA. Despite comparable synovial histopathology, serum MMP-3 levels were significantly higher in patients with D2T RA. MMP-3 levels may reflect ongoing tissue remodelling, cumulative joint damage, or low-grade residual inflammation that is not fully captured by conventional histopathological scoring. In exploratory analyses, inflammatory D2T RA showed higher RSs and MMP-3 levels than noninflammatory D2T RA, supporting the heterogeneity within D2T RA. However, these findings should be interpreted cautiously given their exploratory nature. Future work should explore whether integrating histological assessment with biomarker profiling could improve disease stratification in patients with D2T RA. Importantly, the stratification of inflammatory and noninflammatory D2T RA was performed retrospectively based on the objective clinical parameters observed at the time of surgery and it should therefore be regarded as post hoc and mostly hypothesis generating.

A trend towards a lower RS in patients treated with IL-6 receptor inhibitors than in those receiving abatacept or TNF inhibitors was also observed, although these differences were not statistically significant. It should be emphasised that this analysis may be confounded by treatment selection bias: IL-6 inhibitors are often prescribed to patients with high systemic inflammation, rapid radiographic progression, or prior DMARD failure. Such baseline differences could attenuate the apparent histological advantage of the IL-6 blockade. Prospective stratified comparisons are needed to clarify whether IL-6 inhibition confers superior histological control.

Finally, our study population was limited to surgical cases, which may represent patients with more severe or refractory disease. This selection bias may limit the generalisability of our findings to the broader RA population. In particular, it remains uncertain whether the histological improvements observed here mirror those observed in patients undergoing synovial biopsy or managed without surgical intervention. Validation in nonsurgical cohorts is required to establish the broader applicability of our conclusions.

This study is associated with several limitations that should be acknowledged. First, because the analysis was restricted to surgically obtained synovial specimens, the study population likely represented patients with more advanced or refractory disease, which may limit the generalisability of the findings to broader RA populations. Second, the retrospective study design and lack of standardised treatment duration or timing before surgery may have introduced some heterogeneity in the clinical and pathological assessments. Third, the histological evaluation was performed by a single experienced pathologist to ensure consistency; however, the interobserver reproducibility and blinded repeat scoring were not assessed. In addition, ultrasonographic PD assessment was not performed in a blinded manner, which may have introduced some observer-related bias. Fourth, because multiple synovial specimens from the same patient were included, the assumption of independence between observations may not have been fully met, and within-patient correlations may thus have influenced the results. Finally, no propensity score matching or other advanced adjustment methods were applied, and residual confounding factors could not be excluded, despite the large sample size.

Despite these limitations, this study provides robust evidence that bDMARD therapy leads to measurable histological improvement in RA. Our findings support the potential role of synovial histopathology in guiding personalised therapeutic strategies for RA and emphasise the need for further investigation of the molecular correlates of histological phenotypes.

## CONCLUSION

This study demonstrated that bDMARD therapy suppresses synovial inflammation in RA, as evidenced by the significantly lower RSs in treated patients than in bDMARD-naïve patients. Importantly, synovial inflammation was not significantly different between patients with D2T and those with non-D2T RA, whereas MMP-3 levels were higher in the D2T group. Integrating local tissue-level assessments with biomarker profiling may improve disease stratification and support individualised treatment strategies for patients with RA.

## CRediT authorship contribution statement

**Masanori Sudo:** Writing – review & editing, Writing – original draft, Visualization, Validation, Supervision, Resources, Project administration, Methodology, Investigation, Formal analysis, Data curation, Conceptualization. **Asami Abe:** Writing – review & editing. **Hajime Ishikawa:** Writing – review & editing. **Sayuri Takamura:** Writing – review & editing. **Daisuke Kobayashi:** Writing – review & editing. **Kiyoshi Nakazono:** Writing – review & editing. **Akira Murasawa:** Writing – review & editing. **Suguru Yamamoto:** Writing – review & editing. **Satoshi Ito:** Writing – review & editing.

## Competing interests

All authors declare they have no competing interests.

## Appendix Supplementary materials

Image, application 1

Image, application 2

Image, application 3

## Footnotes

Supplementary material associated with this article can be found, in the online version, at doi:10.1016/j.ero.2026.02.008.

## References

[1] Smolen J.S., Aletaha D., McInnes IB. (2016). Rheumatoid arthritis. Lancet.

[2] Singh J.A., Saag K.G., Bridges S.L., Akl E.A., Bannuru R.R., Sullivan M.C. (2016). 2015 American College of Rheumatology guideline for the treatment of rheumatoid arthritis. Arthritis Rheumatol.

[3] van Vollenhoven RF. (2019). Treat-to-target in rheumatoid arthritis - are we there yet?. Nat Rev Rheumatol.

[4] Taylor P.C., Moore A., Vasilescu R., Alvir J., Tarallo M. (2016). A structured literature review of the burden of illness and unmet needs in patients with rheumatoid arthritis: a current perspective. Rheumatol Int.

[5] Dennis G., Holweg C.T., Kummerfeld S.K., Choy D.F., Setiadi A.F., Hackney J.A. (2014). Synovial phenotypes in rheumatoid arthritis correlate with response to biologic therapeutics. Arthritis Res Ther.

[6] Rooney M., Whelan A., Feighery C., Bresnihan B. (1988). Analysis of the histologic variation of synovitis in rheumatoid arthritis. Arthritis Rheum.

[7] Abe A., Ishikawa H., Nakazono K., Murasawa A., Wakaki K. (2016). A comparison of the ultrasonography images of the joints of patients with rheumatoid arthritis and the corresponding synovial histological findings. Mod Rheumatol.

[8] Nagy G., Roodenrijs N.M.T., Welsing P.M.J., Kedves M., Hamar A., van der Goes M.C. (2021). EULAR definition of difficult-to-treat rheumatoid arthritis. Ann Rheum Dis.

[9] Aletaha D., Neogi T., Silman A.J., Funovits J., Felson D.T. (2010). Bingham CO 3rd, et al. 2010 rheumatoid arthritis classification criteria: an American College of Rheumatology/European League Against Rheumatism collaborative initiative. Ann Rheum Dis.

[10] Larsen A., Dale K., Eek M. (1977). Radiographic evaluation of rheumatoid arthritis and related conditions by standard reference films. Acta Radiol Diagn (Stockh).

[11] Terslev L., Naredo E., Aegerter P., Wakefield R.J., Backhaus M., Balint P. (2017). Scoring ultrasound synovitis in rheumatoid arthritis: a EULAR-OMERACT Ultrasound Taskforce-part 2: reliability and application to multiple joints of a standardised consensus-based scoring system. RMD Open.

[12] Kanda Y. (2013). Investigation of the freely available easy-to-use software “EZR” for medical statistics. Bone Marrow Transplant.

