# Supplementary material for: Synovial histopathology in rheumatoid arthritis treated with biological disease-modifying antirheumatic drugs: an analysis of 1593 surgical specimens using the Rooney score
Source: EULAR Rheumatol Open. 2026 Mar 10;2(1):336–43. doi: 10.1016/j.ero.2026.02.008 (PMC13292280; doi:10.1016/j.ero.2026.02.008)
Supplement: Supplementary file 1 [file mmc1.docx]

**Supplementary Figure S1**


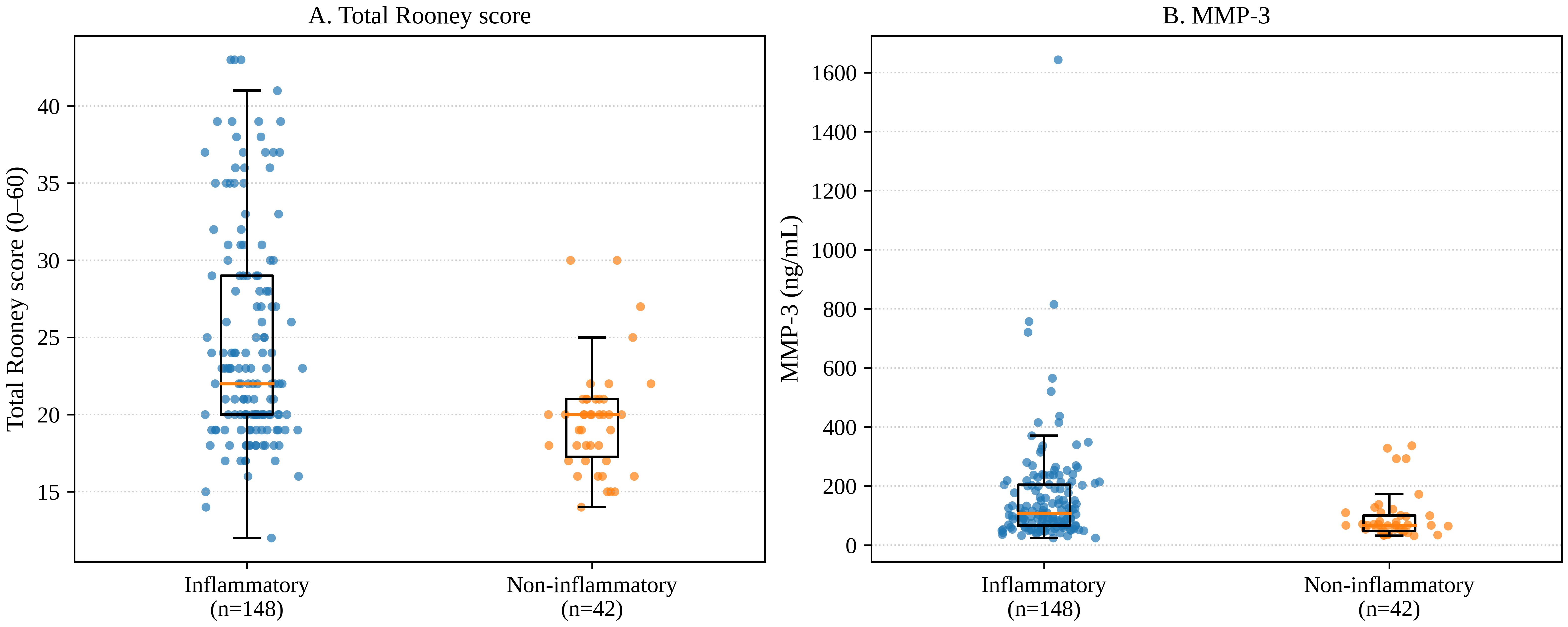


Supplementary Figure S1. Distribution of total Rooney score and MMP-3 levels between inflammatory and non-inflammatory D2T RA according to the liberal rule (CRP >0.3 mg/dL, DAS28-ESR ≥3.2, or PD grade ≥1). Box plots indicate medians and interquartile ranges with individual data points overlaid. Both total Rooney score and MMP-3 levels were significantly higher in the inflammatory group (Mann–Whitney U test, p < 0.001).
