# Supplementary material for: Synovial histopathology in rheumatoid arthritis treated with biological disease-modifying antirheumatic drugs: an analysis of 1593 surgical specimens using the Rooney score
Source: EULAR Rheumatol Open. 2026 Mar 10;2(1):336–43. doi: 10.1016/j.ero.2026.02.008 (PMC13292280; doi:10.1016/j.ero.2026.02.008)
Supplement: Supplementary file 2 [file mmc2.docx]

**Supplementary Table S1. Patient characteristics of bDMARD-treated patients with D2T and non-D2T RA**

| Item | non-D2T RA (n = 393) | D2T RA (n = 190) | P value |
| --- | --- | --- | --- |
| Age (years) | 66 (57–72) | 65 (54–72) | 0.670 |
| Sex (Female, n (%)) | 357 (90.8) | 166 (87.4) | 0.251 |
| Disease duration (years) | 16 (9–23) | 17 (11–23) | 0.305 |
| PSL (mg/day) | 2 (0.0–4.0) | 2 (0.0–3.5) | 0.493 |
| MTX (mg/week) | 4 (0–8) | 0 (0–6) | <0.001* |
| SASP (mg/day) | 1000 (1000–1000) | 1000 (500–1000) | 0.013* |
| BUC (mg/day) | 200 (100–200) | 200 (100–200) | 0.269 |
| IGU (mg/day) | 50 (25–50) | 50 (25–50) | 0.464 |
| TAC (mg/day) | 2 (1–3) | 2 (1–3) | 0.687 |
| MZR (mg/week) | 450 (300–450) | 300 (200–600) | 0.149 |
| Duration of bDMARD therapy (years) | 3 (1–6) | 6 (4–10) | <0.001* |
| Number of bDMARDs used (n) | 1 (1–1) | 3 (2–3) | <0.001* |
| PD grade ≥1 (n, %) | 287 (73.0%) | 129 (67.9%) | 0.235 |
| ACPA (U/mL) | 93.5 (18.2–343.7) | 93.2 (13.9–313.3) | 0.693 |
| CRP (mg/dL) | 0.10 (0.00–0.40) | 0.03 (0.00–0.36) | 0.090 |
| DAS28-ESR | 3.18 (2.61–3.97) | 3.04 (2.07–4.12) | 0.091 |
| MMP-3 (ng/mL) | 79.9 (52.1–135.3) | 95.6 (59.5–190.9) | 0.008* |
| Larsen grade | 4 (3–4) | 4 (3–4) | 0.740 |

Values are presented as the median (interquartile range) or number (%).

P values were calculated using the Mann–Whitney U test for continuous variables and the chi-square test for categorical variables. *Statistically significant (p < 0.05).

Abbreviations: PSL, prednisolone; MTX, methotrexate; SASP, salazosulfapyridine; BUC, bucillamine; IGU, iguratimod; TAC, tacrolimus; MZR, mizoribine; D2T RA, difficult-to-treat rheumatoid arthritis; PD, power Doppler; ACPA, anti-citrullinated protein antibody; CRP, C-reactive protein; DAS28-ESR, Disease Activity Score 28–erythrocyte sedimentation rate; MMP-3, matrix metalloproteinase-3.
