# Supplementary material for: Synovial histopathology in rheumatoid arthritis treated with biological disease-modifying antirheumatic drugs: an analysis of 1593 surgical specimens using the Rooney score
Source: EULAR Rheumatol Open. 2026 Mar 10;2(1):336–43. doi: 10.1016/j.ero.2026.02.008 (PMC13292280; doi:10.1016/j.ero.2026.02.008)
Supplement: Supplementary file 3 [file mmc3.docx]

**Supplementary Table S2. Exploratory comparison of inflammatory vs non-inflammatory D2T RA (liberal rule)**

| Variable | Inflammatory D2T (n=148) | Non-inflammatory D2T (n=42) | p-value |
| --- | --- | --- | --- |
| Total Rooney score (0–60) | 22.00 (20.00-29.00) | 20.00 (17.25-21.00) | <0.001* |
| CRP (mg/dL) | 0.10 (0.01-0.58) | 0.01 (0.00-0.02) | <0.001* |
| MMP-3 (ng/mL) | 107.50 (66.28-204.72) | 67.20 (47.95-100.38) | <0.001* |
| DAS28-ESR | 3.54 (2.33-4.45) | 1.99 (1.55-2.27) | <0.001* |
| PD grade (0–3) | 1.00 (1.00-2.00) | 0.00 (0.00-0.00) | <0.001* |

Inflammatory D2T RA was defined by the presence of objective evidence of inflammation at surgery (CRP >0.3 mg/dL, DAS28-ESR ≥3.2, or PD grade ≥1). Values are presented as the median (interquartile range). P values were calculated using the two-sided Mann–Whitney U test. *Statistically significant (p < 0.05).

Abbreviation: PD, power Doppler; CRP, C-reactive protein; DAS28-ESR, Disease Activity Score 28–erythrocyte sedimentation rate; MMP-3, matrix metalloproteinase-3.
